# Elevated concentrations of methyl isocyanate and isocyanic acid in cigarette smoke

**DOI:** 10.1007/s11356-025-36344-0

**Published:** 2025-04-05

**Authors:** Gunilla Runström Eden, Anders Johansson, Håkan Tinnerberg, Kjell Torén, Daniel Karlsson, Lena Andersson

**Affiliations:** 1 Institute of Medicine, School of Public Health and Community Medicine, University of Gothenburg, Medicinaregatan 16. Box 414, 405 30 Gothenburg, SE Sweden; 2 Department of Occupational and Environmental Medicine, Faculty of Medicine and Health, Örebro University, SE-701 82 Örebro, Sweden; 3 EHS Analytics AB, Staffanstorp, Sweden; 4 Department of Occupational and Environmental Medicine, Örebro University Hospital, 701 85 Örebro, Sweden

**Keywords:** Monoisocyanate, Methyl isocyanate, Isocyanic acid, Exposure, Smoking, Cigarette, Occupational exposure, Easysampler, Impinger

## Abstract

Monoisocyanates are usually intermediates of industrial processes but alkyl-isocyanates and isocyanic acid can also be created when nitrogen-containing compounds thermally degrade. One of the most notable monoisocyanates, methyl isocyanate (MIC), was accidentally released to the surrounding from a pesticide plant in the Bhopal disaster of 1984, where it killed several thousand and injured many more (Mishra et al. Int J Occup Med Environ Health 22(3):193-202 2009). MIC is known to have adverse effects on the respiratory system and other mucous membranes. Links between cigarette smoking and exposure to monoisocyanates have been suggested but not properly evaluated. The aim of the present study was to measure isocyanates in direct smoke and exhaled smoke to determine the potential exposure for the smoker and for people inhaling secondhand smoke. Concentrations of isocyanates were determined in direct cigarette smoke from three different generic cigarette brands using Supelco easysampler ASSET EZ4-NCO and impingers with dibutylamine solution in toluene. Exhaled smoke was measured using easysamplers only and one cigarette brand. Both isocyanic acid (ICA) and MIC were found in direct cigarette smoke at high concentrations (MIC 965–12144 µg/m^3^, ICA 943–5729 µg/m^3^) using either sampling system. Ethyl isocyanate (EIC) and n-propyl isocyanate (PIC) could also be quantified in direct smoke in all three cigarette brands but at much lower concentrations. Measurements of exhaled smoke found MIC in one sample (5.3 µg/m^3^) but no quantifiable levels of ICA or other monoisocyanates. Direct inhalation of cigarette smoke leads to high exposure for ICA and MIC, which exceeds levels set for occupational exposure after only a few cigarettes.

**Graphical abstract:**

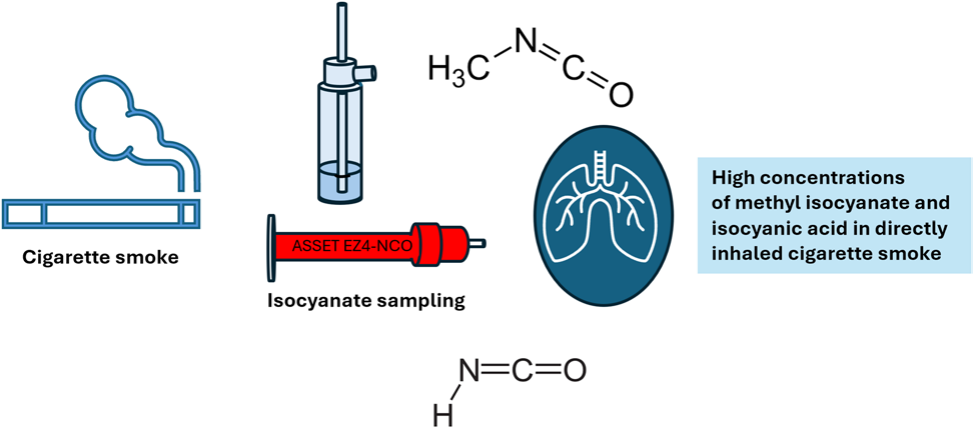

**Supplementary Information:**

The online version contains supplementary material available at 10.1007/s11356-025-36344-0.

## Introduction

Inhalation exposure to methyl isocyanate (MIC) is known to give adverse effects that damage the lining of the respiratory tract. In the Bhopal disaster 1984, extremely high concentrations of MIC were released, causing thousands of deaths and acute and chronic diseases in a large number of people. Pulmonary conditions that have been linked to MIC exposure include chronic obstructive pulmonary disease, emphysema, and fibrosis (Mishra et al. 2009). While diisocyanates are known to cause isocyanate asthma, the same relationship has not been found for MIC (Montelius 2002). MIC, however, has directly irritative effects on mucous membranes and the eyes and high enough doses can cause bronchial lesions and pulmonary edema (Mehta et al. 1990).

One paper from the 1960s has shown that cigarette smoking can be a source of exposure to MIC (Philippe and Honeycutt 1965) with concentrations ranging between 3 and 5 µg/cigarette. Other, more recent, research also suggests a link between cigarette smoking and MIC exposure (Kenwood et al. 2021). Methylcarbamoyl mercapturic acid is a nonselective metabolite of MIC, and concentrations of this in urine in the US population were strongly associated with cigarette smoke exposure (Kenwood et al. 2021). Links were also seen between methylcarbamoyl mercapturic acid concentrations and secondhand smoking. Measurements of cigarette smoke were however not made. Another monoisocyanate, isocyanic acid (ICA), has also been detected in cigarette smoke, but in emission studies (Roberts et al. 2011, Hems et al. 2019) and pyrolysis (Baker and Bishop 2004) and not in direct smoke. Hems et al. (2019) measured ICA in side stream smoke emitted from cigarettes in an environmental chamber. Emissions were presented as a ratio between ICA and carbon monoxide (ppb ICA/ppb CO) and the measurements from cigarette smoke fell into the same range as emission ratios during biomass burning. Roberts et al. (2011) were unable to quantify ICA concentrations as they were too high for their instrument but estimated that the smoking of one cigarette would lead to an inhalation of 36 µg ICA based on the figures from Baker and Bishop (2004). Consequently, a more detailed study is of interest. ICA formation in cigarette smoke can be attributed to oxidation of amines, nicotine, or amides via hydroxyl radicals (Borduas et al. 2013, 2015, 2016).

Health effects of exposure to ICA are less well researched (Montelius 2002). Questionnaire data on foundry workers exposed to ICA and MIC revealed increased ocular and respiratory symptoms compared to controls but effects could not be directly linked to the monoisocyanate exposures (Löfstedt et al. 2009). Exposure to ICA has been linked to a process called carbamylation which involves binding of ICA to proteins, which causes post-translational alterations and malfunctions (Jaisson et al. 2018, Leanderson et al. 2019). Carbamylation of proteins have been implicated in inflammatory diseases (Mydel et al. 2010), chronic renal disease (Kalim et al. 2021), artherosclerosis (Mydel et al. 2010), cardiovascular disease (Wang et al. 2007; Verbrugge et al. 2015), and cataract formation (Liu and Li 1993).

The aim of the present study was to perform an updated measurement of isocyanates in cigarette smoke to determine the exposure to monoisocyanates for the smoker, and to some extent secondary exposure for non-smokers. It is well established that cigarette smoke is harmful to the user, but the role of isocyanate exposure is less investigated. Herein lies the novelty of the research.

Apart from smokers, the results may also be important to personnel who have work tasks that might entail exposure to secondhand cigarette smoke, e.g., home health care or domestic services, as some monoisocyanates have occupational exposure limits. MIC exposure limits vary from 20 to 50 µg/m^3^ (GESTIS substance database). Sweden is the only country in the world to have an occupational exposure limit for isocyanic acid (18 µg/m^3^ 8-h exposure limit, 36 µg/m^3^ short-term exposure limit). This regulation stems from research performed by Karlsson et al. (2001), who showed that high concentrations of ICA and MIC are released during thermal breakdown of polyurethane-containing products. Both substances received occupational exposure limits at the same time, but little information exist on the potential health effects of ICA.

## Materials and methods

### Feasibility study

A feasibility study was conducted before the presented research to determine if isocyanates could be measured in direct smoke using the experimental setup. One cigarette brand was tested in the same experiment location and with a similar setup for direct smoke, using easysampler and impinger. High concentrations of MIC and ICA were present in direct smoke as well as ethyl isocyanate (EIC) and n-propyl isocyanate (PIC) in low concentrations. Isocyanates were also measured in side streams smoke but these were below detection limits for the sampling method (unpublished data). This led to the design of the present study, which focused on direct smoke and exhaled smoke.

### Study area

Tests were carried out in a conservatory attached to a domestic building. The confined space was ca 3 × 8 m with glass doors connected to the outside that could be opened and closed. Roof height was ca 2.5 m. There was no mechanical ventilation. The room was ventilated before and after each experiment to air out the emissions but otherwise the doors remained closed. A particle counter (P-trak 8525, TSI, Minnesota, USA) was used to track emissions and ensure that the room was well-ventilated before the start of the experiment. All direct smoke experiments were conducted at the same time and exhaled smoke experiments were conducted in the same location but at a different time.

### Direct smoke

Isocyanates in direct smoke were measured using two different methods: Supelco easysampler ASSET EZ4-NCO (Merck, Darmstadt Germany) and an impinger sampling system. Cigarettes were directly attached to the samplers via tubes (silicone tubing 6 × 10 mm, VWR) with the aim to sample and quantify the amount of isocyanate that the smoker inhales during smoking. Cigarettes from three different brands were used, one that was considered to be lighter in terms of nicotine content (A), one that was considered stronger (B), and one generic low-cost label (C). Three experiments were done for each cigarette brand using the easysampler. One cigarette for each brand was tested using the impinger system. The order in which the cigarettes were tested was completely randomized.

### Easysampler

**Fig. 1:**
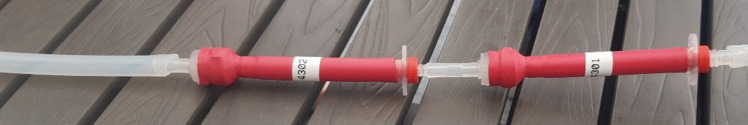

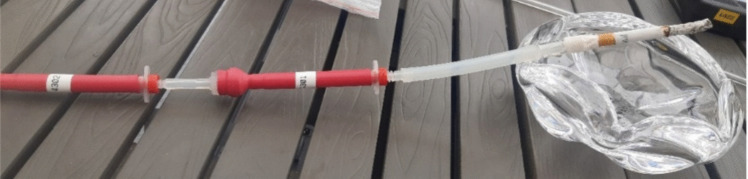
**a** Two connected easysamplers with tubing either end. One tube connects to the cigarette and one to the pump. **b** The connected easysamplers were attached to a cigarette

The easysampler collects isocyanates in both the particular and gaseous phase (Marand et al. 2005) and was used according to the manufacturer’s instructions with minor modifications. Firstly, the flow was increased to 2.0 L/min in order to better represent the inhalation rate of a smoking individual (normal at rest ventilation is 6 L/min, Pleil et al. 2021). Secondly two easysamplers were coupled together in a series. This was done to assert that any breakthrough in the first sampler due to the increased flow would be collected in the second sampler. The flow of 2.0 L/min was the flow when both samplers were connected. Thirdly, the outermost easysampler was connected directly to the cigarette to sample the direct smoke (Fig. 1a and b).

Tape was required to attach the tube to the cigarette for two of the brands but the third brand had a good fit without it. SKC AirChek XR5000 Pumps (SKC, Dorset, UK) were used to draw the air through the samplers. Flow rates were checked before and after the experiment using the TSI series 4100, model 4140 (TSI, Minnesota, USA). Flow rates could only be checked with an unlit cigarette as otherwise smoke would pass through the instrument. Sampling of the direct smoke was done by removing and re-attaching the tube to the pump to simulate a smoker’s inhalation breath. Each inhalation was standardized to last for 5 s and then the tube was removed from the pump for 20 s, before it was attached again for another 5 s. This was repeated until the cigarette was declared to be finished, which was when 1 cm remained before the yellow filter. The number of inhalations that were used was recorded for each cigarette type as well as the start and stop time for each experiment. Easysamplers were closed directly after sampling had finished and kept at refrigerator temperature until analysis. Two field blanks were taken.

Possible losses from using silicone tubing between the pump and the sampler during sampling of ICA and MIC were studied by sampling ICA and MIC in a standard atmosphere. Samplers with 10 cm and 40 cm tubing between the pump and the samplers were parallelly exposed to the standard atmosphere at flow rates of 0.5 and 2.0 l/min. The standard atmosphere of ICA and MIC was generated inside a 300-l stainless steel chamber by thermal degradation of urea and dimethyl urea.

### Impinger sampling system

Isocyanates in direct smoke were also sampled using impingers filled with 10 ml of a 0.01 M dibutylamine solution in toluene according to Karlsson et al. (2001). The impinger also has a filter to collect particulate isocyanates. As with the easysamplers, two different impingers were connected in a series to account for any possible breakthrough during sampling. SKC AirChek XR5000 Pumps (SKC, Dorset, UK) were used to draw the air through the impingers at a flow rate of 2.0 L/min to simulate inhalations and the impingers were in turn connected directly to the cigarette. The standardized inhalation, while the tube was attached to the pump, was set to 3 s instead of 5 to compensate for decreased sampler resistance, with 20 s between each inhalation. The cigarette was declared to be finished when 1 cm remained before the yellow filter. Two field blanks were taken.

### Exhaled smoke

Isocyanates in exhaled smoke were tested using the easysampler and two different collection vessels—a 500-ml round bottom flask with three side necks and a 5000-ml polypropylene Altef gas sampling bag (Restek, CAT22961). Two easysamplers were connected in a series and then attached to a SKC pocket pump 2185 with a flow rate of 0.2 L/min to sample exhaled air. The outermost sampler was used to collect exhaled cigarette smoke from the different collection vessels. For each vessel, a current smoker smoked a cigarette and exhaled each breath into the different vessels. Six exhalations were collected for each experiment. For the round-bottom flask, the easysampler tip was connected to one of the flask openings and measured continuously for 5 min, during which the smoker exhaled into another opening and the third was closed off. The easysampler was held in position in the flask to keep a good a seal as possible but it was not completely airtight. The exhalation into the flask was done by a tube and the seal around the tube into the flask was airtight. The breathing tube was not closed off between exhalations. For the gas sampling bag, six exhalations were made into the bag and the bag was kept closed between exhalations. After the final exhalation, the easysampler tip was connected to the bag and the air was sampled for 10 min. The experiment was done once per collection vessel using one of the generic cigarette brands (Brand C). One field blank was taken.

### Chemical analysis

Analysis was performed by HPLC–MS/MS (UPLC Accela 1250 med Accela Autosampler och TSQ Quantum Access Max med HESI-II detektor) at the Department of Occupational and Environmental Medicine, Örebro University Hospital, Sweden. The dibutylamine solution and the impinger filter were analyzed separately (ISO 17734-1:^2013^; ISO 17734–2:^2013^).

### Particle, carbon dioxide, temperature, and relative humidity measurements

Carbon dioxide, temperature, and relative humidity in the room were measured simultaneously throughout the direct smoke experiments using a CA 1510 logger. During exhaled smoke measurements, a Testo 174H was used, which only measures temperature and relative humidity. Particle emissions were measured using the P-trak 8525 particle counter, which measures particle numbers in the size range of 20 nm–1 µm. The P-trak instrument was placed close to the source and emissions were tracked to ensure that all cigarette smoke had been ventilated out between each measurement. The room was considered to be ventilated when concentrations reached ca 1000–3500 pt/ml, which was equivalent to background levels.

## Results

### Sampling setup

No significant losses of ICA or MIC were observed for flow rates of 0.5 l/min or 2.0 l/min when increasing the length of the tubing from 10 to 40 cm (Online resource Table S1).

### Direct smoke

There was large variation in the number of inhalations that was required to deplete the cigarette and no clear trend was seen for the individual brands (Table 1). The easysamplers produced higher flow resistance than the impinger sampling system and at times strained the pump.

**Table 1:** Monoisocyanate concentrations in direct smoke sampled by easysampler and impinger. Concentrations in series-connected samplers have been added together. For each cigarette brand, the mean easysampler concentration is also displayed. Inhalations were 5 s long for easysampler measurements and 3 s for impinger measurements

Cigarette brand	Sampler	Sample number	Number of inhalations	ICA µg/cigarette µg/m^3^		MIC µg/cigarette µg/m^3^		EIC µg/cigarette µg/m^3^		PIC µg/cigarette µg/m^3^	
A	Impinger		11 (3 s)	3.8	3474	9.2	8323	1.3	1196	0.2	179
	Easysampler	Mean A1-A3	16	2.8	1086	2.6	965	0.6	210	0.2	59
	Easysampler	A:1	15	2.7	1066	3.3	1316	0.7	288	0.2	78
	Easysampler	A:2	16	4.2	1563	2.8	1049	0.6	229	0.2	66
	Easysampler	A:3	18	1.9	628	1.6	530	0.3	112	0.1	34
B	Impinger		10 (3 s)	5.8	5729	12.1	12,144	1.4	1413	0.2	235
	Easysampler	Mean B1-3	17	3.1	1138	3.0	1103	0.6	202	0.1	56
	Easysampler	B:1	17	3.0	1061	3.2	1116	0.6	194	0.2	53
	Easysampler	B:2	14	3.9	1671	3.7	1601	0.7	305	0.2	83
	Easysampler	B:3	21	2.4	684	2.1	591	0.4	108	0.1	33
C	Impinger		14 (3 s)	2.4	1714	8.1	5779	1.1	798	0.2	109
	Easysampler	Mean C1-3	16	2.5	943	3.1	1128	0.7	256	0.2	69
	Easysampler	C:1	16	3.1	1155	3.6	1337	0.9	322	0.2	86
	Easysampler	C:2	16	2.0	765	3.0	1104	0.6	240	0.2	64
	Easysampler	C:3	17	2.6	909	2.7	943	0.6	205	0.2	56

Isocyanic acid (ICA), methyl isocyanate (MIC), ethyl isocyanate (EIC), and n-propyl isocyanate (PIC) could be quantified in direct smoke in all three cigarette brands using both the easysampler and the impinger system. No quantifiable levels of phenylisocyanate, hexamethylene diisocyanate, toluene diisocyanate, methylene diphenyl diisocyanate, or isophoron diisocyanate could be detected in any sample. The quantification limits of the isocyanates were 0.005 µg/sample apart from toluene diisocyanate, which was 0.010 µg/sample and ICA, which was 0.3 µg/sample. Samplers were connected in a series and breakthrough from the first sampler to the second was observed in all easysampler measurements (Online resource Table S2) but not in the impinger sampling system, where only ICA could be determined in all four samples (filter and liquid) (Online resource Table S3).

Concentrations of isocyanic acid and methyl isocyanates were very high (MIC 965–12144 µg/m^3^, ICA 943–5729 µg/m^3^) in the direct smoke using either sampling system (Table 1). Concentrations within the same cigarette brand varied, but measured MIC and ICA levels were similar in the experiments using the easysamplers. Using the impingers, the ratio of MIC to ICA was different, where MIC levels exceeded those of ICA by around 200–300% (Table 1).

### Exhaled smoke

Exhaled smoke measurements only found quantifiable levels of MIC. This was determined using the Altef gas sampling bag, and then only in the first of the connected easysamplers. MIC concentration in that one sample was 5.3 µg/m^3^. The remaining samples were below level of quantification.

### Temperature, carbon dioxide, and relative humidity

Temperature, carbon dioxide levels, and relative humidity in the sampling location did not change considerably during the direct smoke experiments. Temperature remained within 23.7–28 °C, relative humidity within 44.5–54.5% and carbon dioxide levels were between 448 and 496 ppm. During the exhaled smoke experiments, temperature was 20.3 °C and relative humidity was 58%.

## Discussion

The present study has demonstrated that high concentrations of methylisocyanate (MIC) and isocyanic acid (ICA) are produced during cigarette smoking, which are inhaled by the smoker. High concentrations during smoking might therefore be a contributing factor to respiratory disease in smokers, as MIC, in particular, has been linked to adverse respiratory effects (Corrin and Nicholson 2011). MIC concentrations were much lower in exhaled smoke compared to those in direct smoke, indicating a reduced exposure to secondhand smoke. In the exhaled smoke experiments, only exhaled air was sampled, meaning that any addition of MIC from the burning cigarette between smoking was not included. Measurements of side stream smoke in the feasibility study did not detect concentrations of MIC above the detection limit. However, as only one cigarette was smoked, the results might have been different if sampling had been carried out in an environment where cigarette smoking was more frequent, e.g., smoking rooms at airports or in the homes of smokers. Further experiments are needed to determine if secondhand smoke concentrations would reach MIC concentrations of consequence.

ICA was not found at all in exhaled smoke. This could have several reasons. ICA could have interacted with the respiratory tract upon inhalation or there might have been insufficiencies in the experiment setup. In direct smoke, concentrations were high, similar as can be seen in industries that use urea-containing glues (Tinnerberg et al. 2024).

MIC concentrations per cigarette smoked were in line with previously reported values by Philippe and Honeycutt (ca 2–12 µg and 3–5 µg respectively). Roberts et al. (2011) estimated an exposure of 36 µg of ICA for a filtered cigarette based on pyrolysis experiments. However, the present study recorded a tenth of that (ca 2–5 µg). One reason for this discrepancy could be different temperatures of combustion. It has been observed that more ICA is produced at higher temperature combustion than when only smoldering occurs (Roberts et al. 2011).

Low concentrations of ICA can be found in the surrounding air from environmental sources (Leslie et al. 2019, Wang et al. 2022) and therefore it is possible that the concentrations determined in the study could have an additional source from the ambient air. Cooking can be a major source for ICA, and to lesser degree MIC (Leanderson 2019; Wang et al. 2022) but no cooking was carried out during the experiment. Similarly, MIC can be generated from environmental sources, and it has a long atmospheric residence time (Papanastasiou et al. 2016). Long-term measurements would have to be carried out to quantify the additional amount of ICA and MIC that comes from the environmental sources, but it is expected to be very low.

MIC has occupational exposure limits and short-term exposure limits in many countries (GESTIS substance database), whereas Sweden is alone in also having one for ICA. Occupational short-term exposure limits in Sweden are 36 µg/m^3^ for ICA and 47 µg/m^3^ for MIC, for a 5-min average. Comparing the measured concentrations in direct cigarette smoke with short-term exposure limits, it would take less than one cigarette to exceed the exposure limit for MIC and for ICA. Our findings underscore the importance of active smoking as a source of MIC and ICA. Especially, MIC is toxic for the airways via inhalation and may contribute to fibrosis, degeneration, and obstruction in the airways by damaging the respiratory tract cell linings (Varma and Guest 1993; Kenwood et al. 2021).

The study has several limitations. Even with the maximum pump flow that the samplers could handle, the inhalation flow of 2L/min is less than the inhalation flow rate of an actual smoker. It was also impossible to check the flow rate with a lit cigarette attached to the samplers, which means that the true flow rate in the experiments might differ from the preset one. The occasional straining of the pump using the easysamplers suggest an increased resistance in the sampler, which could have affected the flow rate. Increased or decreased flow rate would influence the temperature at which the cigarette burns, which could in turn affect the composition of the combustion gases.

Samplers were connected in series and break through was evident in the easysamplers and to some extent the impingers. Use of nicotine research standard cigarettes could have made the study more comparable with other published research but were found to be practically challenging to obtain. It was decided that for the purpose of the study, generic branded cigarettes would suffice to capture the exposure.

## Conclusion

The present study found that inhaling cigarette smoke exposed the smoker to very high concentrations of MIC and ICA, which could contribute to the adverse health effects from smoking. Only one cigarette was needed to exceed short-term occupational exposure limits. Concentrations in side-stream smoke were considerably lower but needs to be examined further. MIC and ICA concentrations, and potentially flow rates, varied between the experiments and the exact numbers reported, should therefore be regarded with some uncertainty. However, the findings of high levels of MIC and ICA in cigarette smoke are still convincing.

## Supplementary Information

Below is the link to the electronic supplementary material.

Supplementary file1 (DOCX 25 KB)

## Data Availability

Results from the experimental loss study, Easysampler results, and Impinger results are available in the online resource attached to the manuscript. The remaining isocyanate results are presented in the paper.
