# Supplementary material for: Elevated concentrations of methyl isocyanate and isocyanic acid in cigarette smoke
Source: Environ Sci Pollut Res Int. 2025 Apr 5;32(17):11016–23. doi: 10.1007/s11356-025-36344-0 (PMC12014798; doi:10.1007/s11356-025-36344-0)
Supplement: Supplementary file 1 — Supplementary file1 (DOCX 25 KB) [file 11356_2025_36344_MOESM1_ESM.docx]

Table S1. Concentrations of ICA and MIC during parallel sampling using 10 and 40 cm silicone tubing prior to the sampler. Separate tests with different generated amounts of ICA and MIC were performed for the different flow rates.

| Flow rate (l/min) | Tubing length (cm) | ICA (µg/m^3^) | MIC (µg/m^3^) |
| --- | --- | --- | --- |
| 2.0 | 10 | 0.104 | 0.321 |
|  | 40 | 0.111 | 0.319 |
| 0.5 | 10 | 0.328 | 0.060 |
|  | 40 | 0.322 | 0.056 |

Table S2. Easysampler results for all individual samplers. The first position means the position closest to the cigarette. Inhalations refers to the number of inhalations required to deplete the cigarette.

| **Cigarette brand** | **Sampler position** | **Inhalations**  **nr** | **ICA**  **µg** | **µg/m^3^** | **MIC**  **µg** | **µg/m^3^** | **EIC**  **µg** | **µg/m^3^** | **PIC**  **µg** | **µg/m^3^** |
| --- | --- | --- | --- | --- | --- | --- | --- | --- | --- | --- |
| A | 1st | 15 | 2,6 | 1047 | 2,6 | 1040 | 0,51 | 204 | 0,14 | 58 |
| A | 2nd | 15 | 0,05 | 19 | 0,69 | 276 | 0,21 | 84 | 0,05 | 20 |
| A | 1st | 16 | 4,1 | 1542 | 2,1 | 787 | 0,4 | 150 | 0,12 | 46 |
| A | 2nd | 16 | 0,06 | 21 | 0,70 | 262 | 0,21 | 79 | 0,05 | 20 |
| A | 1st | 18 | 1,7 | 573 | 1,3 | 433 | 0,24 | 80 | 0,08 | 27 |
| A | 2nd | 18 | 0,17 | 55 | 0,29 | 97 | 0,095 | 32 | 0,02 | 7 |
| B | 1st | 17 | 2,9 | 1030 | 2,5 | 883 | 0,4 | 141 | 0,11 | 40 |
| B | 2nd | 17 | 0,09 | 31 | 0,66 | 233 | 0,15 | 53 | 0,04 | 13 |
| B | 1st | 14 | 3,8 | 1638 | 2,8 | 1202 | 0,48 | 206 | 0,13 | 58 |
| B | 2nd | 14 | 0,08 | 33 | 0,93 | 399 | 0,23 | 99 | 0,06 | 25 |
| B | 1st | 21 | 2,3 | 662 | 1,6 | 457 | 0,26 | 74 | 0,08 | 24 |
| B | 2nd | 21 | 0,08 | 22 | 0,47 | 134 | 0,12 | 34 | 0,03 | 9 |
| C | 1st | 16 | 3,0 | 1130 | 2,8 | 1049 | 0,62 | 232 | 0,17 | 65 |
| C | 2nd | 16 | 0,07 | 25 | 0,77 | 288 | 0,24 | 90 | 0,06 | 21 |
| C | 1st | 16 | 2,0 | 755 | 2,3 | 861 | 0,44 | 165 | 0,12 | 46 |
| C | 2nd | 16 | 0,03 | 10 | 0,65 | 243 | 0,2 | 75 | 0,05 | 18 |
| C | 1st | 17 | 2,5 | 889 | 1,9 | 671 | 0,36 | 127 | 0,10 | 37 |
| C | 2nd | 17 | 0,06 | 20 | 0,77 | 272 | 0,22 | 78 | 0,05 | 19 |

Table S3. Impinger results. The first position means the position closest to the cigarette. Inhalations refers to the number of inhalations required to deplete the cigarette.

| **Cigarette brand** | **Impinger**  **position** | **Sample**  **Type** | **Inhalations**  **nr** | **ICA**  **µg** | **µg/m^3^** | **MIC**  **µg** | **µg/m^3^** | **EIC**  **µg** | **µg/m3** | **PIC**  **µg** | **µg/m^3^** |
| --- | --- | --- | --- | --- | --- | --- | --- | --- | --- | --- | --- |
| A | 1st | Liquid | 11 | 2,30 | 2089 | 9 | 8182 | 1,3 | 1182 | 0,18 | 164 |
| A | 1st | Filter | 11 | 1,40 | 1270 | 0,04 | 41 | 0,01 | 6 | 0,02 | 15 |
| A | 2nd | Liquid | 11 | 0,09 | 85 | 0,11 | 100 | 0,01 | 8 |  |  |
| A | 2nd | Filter | 11 | 0,03 | 30 |  |  |  |  |  |  |
| B | 1st | Liquid | 10 | 4,59 | 4598 | 12 | 12000 | 1,4 | 1400 | 0,22 | 220 |
| B | 1st | Filter | 10 | 1,14 | 1097 | 0,03 | 34 | 0,01 | 5 | 0,02 | 15 |
| B | 2nd | Liquid | 10 | 0,02 | 19 | 0,11 | 110 | 0,01 | 8 |  |  |
| B | 2nd | Filter | 10 | 0,01 | 15 |  |  |  |  |  |  |
| C | 1st | Liquid | 14 | 1,60 | 1141 | 8 | 5714 | 1,1 | 786 | 0,14 | 100 |
| C | 1st | Filter | 14 | 0,70 | 498 | 0,03 | 19 | 0,01 | 4 | 0,01 | 9 |
| C | 2nd | Liquid | 14 | 0,08 | 59 | 0,06 | 46 | 0,01 | 4 |  |  |
| C | 2nd | Filter | 14 | 0,02 | 16 |  |  |  |  |  |  |
